# Visualizing balances of compositional data: A new alternative to balance dendrograms

**DOI:** 10.12688/f1000research.15858.1

**Published:** 2018-08-14

**Authors:** Thomas P. Quinn

**Affiliations:** 1 Bioinformatics Core Research Group, Deakin University, Geelong, Victoria, 3220, Australia

**Keywords:** compositional data, coda, balances, ilr, visualization, rstats, r

## Abstract

Balances have become a cornerstone of compositional data analysis. However, conceptualizing balances is difficult, especially for high-dimensional data. Most often, investigators visualize balances with the balance dendrogram, but this technique is not necessarily intuitive and does not scale well for large data. This manuscript introduces the 'balance' package for the R programming language. This package visualizes balances of compositional data using an alternative to the balance dendrogram. This alternative contains the same information coded by the balance dendrogram, but projects data on a common scale that facilitates direct comparisons and accommodates high-dimensional data. By stripping the branches from the tree, 'balance' can cleanly visualize any subset of balances without disrupting the interpretation of the remaining balances. As an example, this package is applied to a publicly available meta-genomics data set measuring the relative abundance of 500 microbe taxa.

## Introduction

A composition is a vector of positive measurements that sum to an arbitrary total ^1^. Examples of compositions include measurements recorded in parts per million (ppm) or percentages, but also include measurements that are less obviously parts of the whole (e.g., count data generated by next-generation sequencing ^2^). A component is one part of a composition. Compositional data analysis (CoDA) deals with the analysis of compositions. Compositional data, because they contain values bounded from zero to one, exist in a non-Euclidean space that render conventional statistical methods invalid. To deal with compositionality, CoDA typically begins with a log-ratio transformation that maps data into an unbounded space where conventional statistical methods can be used. The simplest transformations, the centered log-ratio transformation and the additive log-ratio transformation, use a simple reference as the denominator of the log-ratio. A more complex transformation, the isometric log-ratio transformation, transforms the composition with respect to an orthonormal basis ^3^. Alternatively, one could analyze the log-ratio of each component to the other directly ^4, 5^.

Balances use a sequential binary partition (SBP) to define an orthonormal basis that splits the composition into a series of non-overlapping groups ^6^. This design allows for an interpretation of the data at the level of the isometric log-ratio coordinates ^7^. This SBP contains a diverging set of contrasts that are each interpretable as a measure of “Group 1 vs. Group 2” (following an isometric log-ratio transformation). For a *D*-part composition, the SBP defines *D −* 1 balances that decompose the variance such the sum of the sample-wise variances for each balance in the tree equals the total sample-wise variance ^6^. Balances (like the centered log-ratio transformation and the isometric log-ratio transformation) satisfy all properties required for compositional data analysis: scale invariance, permutation invariance, perturbation invariance, and sub-compositional dominance (reviewed in 8 and elsewhere).

Although balances have proved useful for the analysis of compositional data, their usual application depends on generating a meaningful SBP. Sometimes, this involves manually creating an SBP based on expert opinion, with or without the assitance of exploratory analyses ^6^. However, using expertise to build an SBP is not always desirable, especially for high-dimensional data (where each composition can measure thousands of components). Principal balance analysis is a data-driven alternative that, similar to principal component analysis, seeks to identify an SBP whose balances successively explain the maximal variance of a data set (a computationally expensive procedure approximated with heuristics) ^9, 10^. In the field of meta-genomics, where next-generation sequencing is used to count the relative abundance of microbe taxa, scientists have applied balances of SBPs to summarize and classify microbiome samples ^11^. One study defined the SBP by hierarchically clustering the microbe taxa based on the outcome of interest ^12^. Another defined the SBP based on the phylogenetic relationship between microorganisms ^13^.

Once an SBP is generated, its balances can be visualized using a balance dendrogram ^14^. The balance dendrogram illustrates (a) the distribution of samples across the balance, (b) the relationship between balances along the SBP tree, and (c) the decomposition of variance ^6, 15^. In addition, a balance dendrogram can show differences between sub-groupings of samples by coloring facets of the box plots. Although balance dendrograms capture a vast amount of data, the balance dendrogram may not provide the optimal visualization of balances. First, by building the figure around a tree, balance dendrograms place emphasis on the relationship between the balances, and not on the balances themselves. Second, each box plot has a unique scale positioned sporadically along the tree such that direct comparisons between one balance and all others become difficult. Third, the decomposition of variance uses lines that run parallel to the dendrogram branches, potentially confusing these concepts through use of a common symbol. In this software article, I present the R package balance for visualizing balances of compositional data. This package provides an alternative to the balance dendrogram that I hope will simplify balances for scientists less familiar with compositional data analysis.

## Methods

### Implementation

Within the R package universe, there are three standalone and well-documented tools for general compositional data analysis: compositions ^16^, robCompositions ^17^, and zCompositions ^18^. The compositions::CoDaDendrogram function plots an archetypal balance dendrogram. There are also a number of domain-specific tools, tailored to next-generation sequencing data, and shown to work effectively ^19, 20^: ALDEx2 ^21, 22^ and ANCOM ^23^ for differential abundance analysis, SparCC ^24^ and SPIEC-EASI ^25^ for the correlation analysis of sparse networks, propr ^26, 27^ for proportionality analysis, and philr ^13^ for the analysis of phylogeny-based balances. Of these, the philr package computes balances and visualizes them with dendrograms, but does not plot a balance dendrogram *per se*.

The balance package is available for the R programming language and uses ggplot2 ^28^ to visualize the distribution of samples across balances of a sequential binary partition (SBP) matrix. Each balance is calculated by the formula:

$$b_{i}=\sqrt{\frac{\left|i_{p}\right|\left|i_{n}\right|}{\left|i_{p}\right|+\left|i_{n}\right|}}\;\log\left[\frac{g\left(i_{p}\right)}{g\left(i_{n}\right)}\right]$$

for *b _i_* = [ *b*_1_ *, ..., b _D−_*_1_] balances where *g*( *x*) is the geometric mean of *x*, *i _p_* is the sub-composition of *positively*-valanced components, and *i _n_* is the sub-composition of *negatively*-valanced components. Here, | *i _p_*| describes the norm, or length, of the sub-composition.

### Operation

The balance package ^29^ computes and visualizes balances of compositional data. It requires few package dependencies, has negligible system requirements, and runs fast on a standard laptop computer (e.g., any modern budget CPU with 4GB RAM). To use balance, the user must provide a compositional data set (e.g., Table 1: samples as rows and components as columns) and a serial binary partition (SBP) matrix (e.g., Table 2: components as rows and balances as columns). Below, balance is shown for an example data set from robCompositions ^17^.

library(robCompositions)
library(balance)
data(expenditures, package = "robCompositions")
y1 <− data.frame(c(1,1,1,−1,−1),c(1,−1,−1,0,0),
                    c(0,+1,−1,0,0),c(0,0,0,+1,−1))
res <− balance(expenditures, y1)

**Table 1.** An example of a compositional data set with 20 sample compositions measuring 5 components each. As compositional data, the total expenditure for each individual is arbitrary. These example data are taken from robCompositions ^17^.

housing	foodstuffs	alcohol	other	services
640	328	147	169	196
1800	484	515	2291	912
2085	445	725	8373	1732
616	331	126	117	149
875	368	191	290	275
770	364	196	242	236
990	415	284	588	420
414	305	94	68	112
1394	440	393	1161	636
1285	374	363	785	487
1102	469	243	496	388
1717	452	452	1977	832
1549	454	424	1345	676
838	386	155	208	222
845	386	211	317	280
1130	394	271	490	386
1765	466	524	2133	822
1195	443	329	974	523
2180	521	553	2781	1010
1017	410	225	419	345

**Table 2.** An example of a serial binary partition (SBP) matrix with 5 components split into 4 balances. These example data are taken from robCompositions ^17^.

z1	z2	z3	z4
1	1	0	0
1	-1	1	0
1	-1	-1	0
-1	0	0	1
-1	0	0	-1

Optionally, users can color components or samples based on user-defined groupings. To do this, users must provide a vector of group labels for each component via the d.group argument (or for each sample via the n.group argument). The boxplot.split argument facets the box plots similar to the balance dendrogram ^15^.

group <− c(rep("A", 10), rep("B", 10))
res <− balance(expenditures, y1, n.group = group, boxplot.split = TRUE)

**Figure 1.**
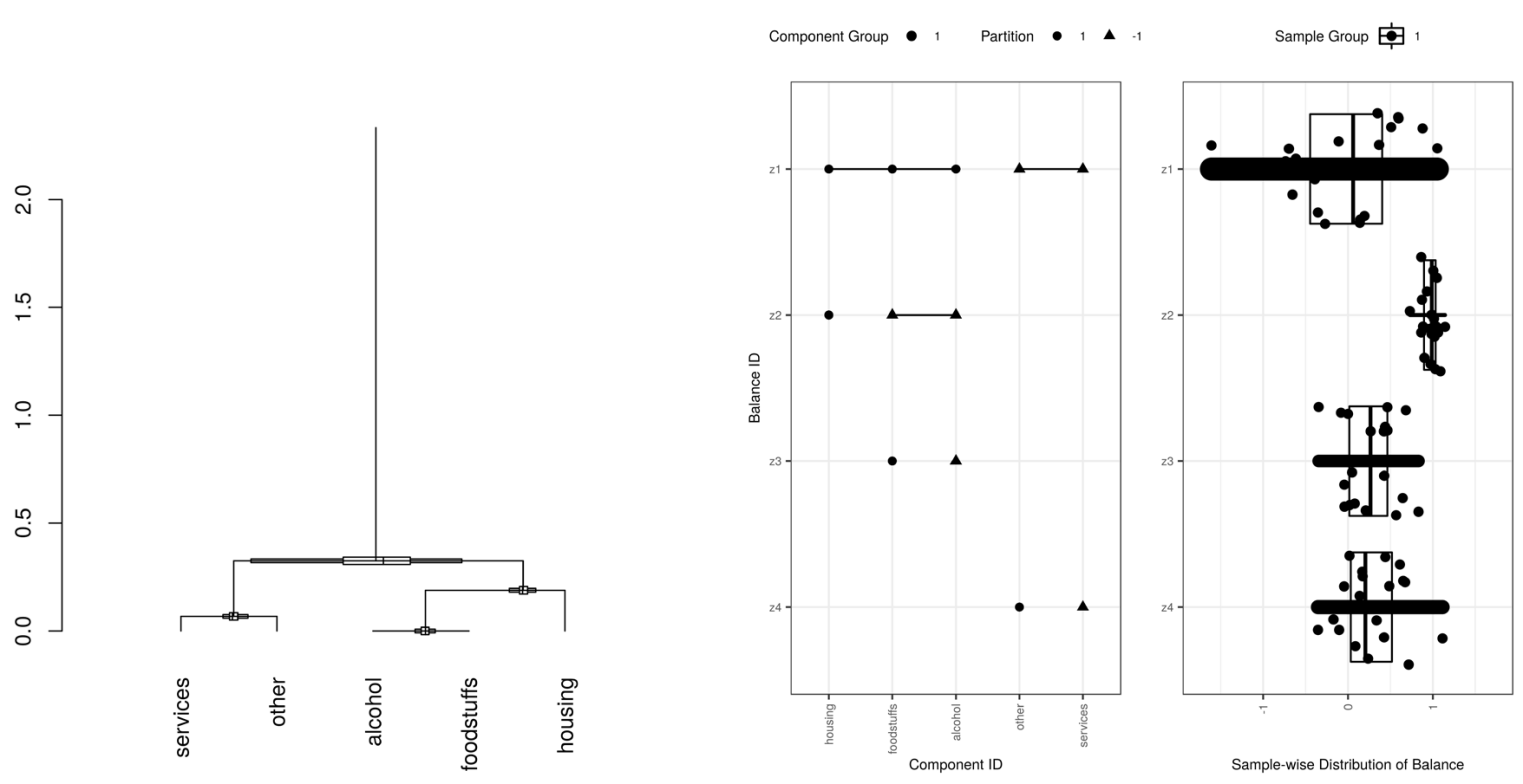
This figure shows a balance dendrogram and its alternative, both prepared using the data from Table 1 and Table 2. On the left, first branch of the balance dendrogram shows how the “services” and “other” components are contrasted against the remaining components. The box plot positioned at the branch shows the distribution of samples within this balance. The length of trunk shows the proportion of variance explained by this balance. On the right, this same information gets captured by a two-panel figure. The top balance in the left panel shows how the “services” and “other” components are contrasted against the remaining components. The top balance in the right panel shows the distribution of samples within this balance. In the right panel, the line length shows the range of the sample distribution, while its thickness shows the proportion of variance explained. Note that the median of this first contrast sits slightly positive, meaning that the most samples spend more on [“alcohol”, “foodstuff”, “housing”].

Figure 1 compares the balance dendrogram to its alternative using the robCompositions data ^17^.

## Use cases

As a use case, a publicly available microbiome data set is analyzed using balances. These data measure the abundance of microbe taxa in the feces of diabetics and their non-diabetic relatives ^30^, making it a true relative data set. Since these data contain many zeros that disrupt the log-ratio transformations, the zeros are first replaced through imputation by the zCompositions package. See the Supplementary Information for a demonstration of other pre-processing steps.

To identify balances for visualization, a serial binary partition (SBP) matrix is made by hierarchically clustering components based on their proportionality measure *ϕ _s_* (used here as a dissimilarity measure ^27^), thus joining together components that covary similarly across all samples. The ape ^31^ and philr ^13^ packages transform the tree object into an SBP ready for analysis and visualization.

# for compositional data with samples as rows
data.no0 <− zCompositions::cmultRepl(data, method = "CZM")
pr <− propr::propr(data.no0, metric = "phs")
h <− hclust(as.dist(pr@matrix))
phylo <− ape::as.phylo(h)
sbp <− philr::phylo2sbp(phylo)
# it is helpful to name the balances
colnames(sbp) <− paste("z", 1:ncol(sbp))
res <− balance::balance(data.no0, sbp, size.text = 4,size.pt = 1)

**Figure 2.**
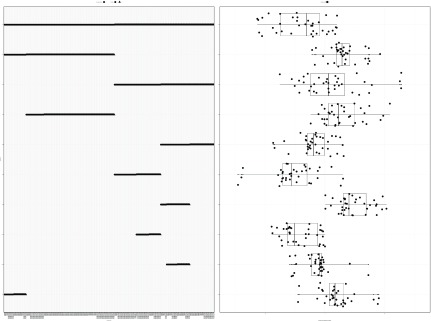
This figure shows the top 10 most explanatory balances, ranked by the proportion of variance explained. The left panel shows how select microbe taxa are contrasted against others. The right panel shows the corresponding distribution of samples within each balance, with the line length showing the range of the distribution. Many of the most explanatory balances occur toward the base of the serial binary partition (SBP) matrix. Yet, this subset visualization is not feasible with the balance dendrogram. Note that the order among the top 10 balances is determined procedurally to place the base of the tree at the top of the figure.

Supplementary Figure 1 visualizes all 499 balances and contains the same information that a balance dendrogram would contain: (a) the left panel dot plot shows the components being contrasted, (b) the right panel box plot shows the distribution of samples across each balance, and (c) the right panel line length shows the range of the balance (the range should cleanly approximate the decomposition of variance for purpose of exploratory visualization, though line width can optionally show the actual proportion of explained variance if desired). However, unlike a balance dendrogram, components and samples are projected on a common scale that facilitates direct comparisons and accommodates high-dimensional data. Yet, the main advantage of the balance package is that, by stripping the branches from the tree, it becomes possible to visualize any subset of balances without disrupting the interpretation of the remaining balances. In Figure 2, we subset the visualization to include only the top 10 most explanatory balances, ranked by the proportion of variance explained.


# full balances stored in results of balance plot
balances <− res[[3]]
vars <− apply(balances, 2, var)
rank <− order(vars, decreasing = TRUE)[1:10]
res <− balance::balance(data.no0, sbp[,rank], size.text = 4)
# then view for further study
sbp[,rank]

**Figure 3.**
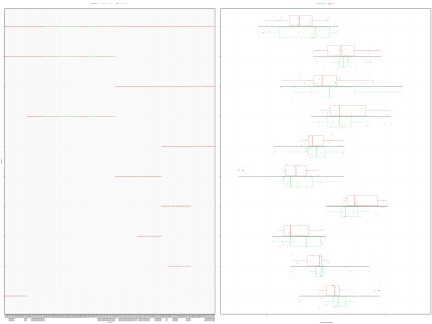
This figure shows the top 10 most explanatory balances, ranked by the proportion of variance explained, with points colored by the user-defined groupings. The left panel shows how select microbe taxa are contrasted against others. The right panel shows the corresponding distribution of samples for each group within each balance, with the line length showing the total range of the distribution. There is apparently a difference in the median values of diabetics and non-diabetics for some balances. One could test the significance of these differences using conventional statistical methods like the Student’s *t*-test ^32^. Note that the order among the top 10 balances is determined procedurally to place the base of the tree at the top of the figure.

The d.group and n.group arguments offer a way to organize the results in a meaningful way. For example, the d.group can label microbes that most interest investigators, while the n.group can label patients based on clinical findings. Here, colored components ( d.group) indicate the availability of supplemental meta-transcriptomic data, while colored samples ( n.group) indicate the presence or absence of type-1 diabetes. In Figure 3, we repeat the visualization of the top 10 most explanatory balances, with points colored by the user-defined groupings.

## Summary

Compositional data measure parts of a whole such that the total sum of the composition is irrelevant and each part is only interpretable relative to others. The analysis of composition data requires interpreting the parts of the composition relative to the others. Log-ratio transformations offer a way to transform the data into an unbounded space where the analyst can apply conventional statistical methods. One transformation is the isometric log-ratio transformation which transforms the composition with respect to an orthonormal basis. Balances use a sequential binary partition (SBP) to define an orthonormal basis that splits the composition into a series of non-overlapping groups. Balances can help the investigator identify trends in relative data, and are often visualized using a balance dendrogram. However, the balance dendrogram is not necessarily intuitive and does not scale well for large data. This paper introduces the balance package for the R programming language, a package for visualizing balances of compositional data using an alternative to the balance dendrogram. This alternative contains the same information coded by the balance dendrogram, but projects data on a common scale that facilitates direct comparisons and accommodates high-dimensional data. By stripping the branches from the tree, balance can cleanly visualize any subset of balances without disrupting the interpretation of the remaining balances.

## Data availability

All data used for this analysis were acquired from the supplement of Heintz-Buschart *et al*. ^30^. The supplement of this manuscript contains code to pre-process these data and reproduce the analysis.

## Software availability

Software and source code available from: https://github.com/tpq/balance

Archived source code at time of publication: https://doi.org/10.5281/zenodo.1326860 ^29^

Software license: GPL-2

## Supplementary material

Supplementary Information. All data and scripts needed to reproduce the analysis.

Supplementary Figure 1. Visualization of all 499 balances of the example microbial taxa data set. While this figure contains the same information as a balance dendrogram, it projects data on a common scale that facilitates direct comparisons and accommodates high-dimensional data.
