# Supplementary material for: Visualizing balances of compositional data: A new alternative to balance dendrograms
Source: F1000Res. 2018 Aug 14;7:1278. [Version 1] doi: 10.12688/f1000research.15858.1 (PMC6124381; doi:10.12688/f1000research.15858.1)
Supplement: Peer review history

# Peer review: Referee response for version 1

**DOI:** 10.5256/f1000research.17311.r37158

**Published:** 2018-09-03

**Authors:** Marc Noguera-Julian

**Affiliations:** 1 IrsiCaixa AIDS Research Institute, Badalona, Spain; 2 Universitat de Vic-Universitat Central de Catalunya, Catalonia, Spain

Manuscript by Quinn entitled “Visualizing balances of compositional data” introduces a new way to visualize balances and component partitions in compositional data. Typically, these are visualized using dendrograms where branches represent the component partitions (obtained through an external method and/or expert knowledge). The height of the branching point in the y-axis represents the proportion of variance explained, while intersection on the branching point represents the mean of the balance. Structure of the dendrogram relates to the partition hierarchy. In addition, boxplots can be defined on top of the dendrogram, at each branching point, depicting the distribution of balance values. These dendrograms are useful when a bunch of variables are analyzed but are hardly interpretable when in a high-dimensional space, which is often the case in omics derived data.

 At present, the interpretation of CoDA results in terms of clinical/biological meaning is one of the bottlenecks for the adoption of such theoretical frameworks in omics data-based clinical research. In this context, new ideas on CoDA visualization are welcome and useful.

 Quinn’s proposal splits the data that characterizes balances into two parts, one regarding the partition of the components into balances (left sub-plot) and the other to the distribution of the balances values when applied to the compositional data and the distribution of the variance of the same data (right sub-plot).This is similar to moving the boxplots aside from the dendrogram and turning the dendrogram into a simple sequential partition diagram. In addition, a grouping factor can be projected into the right sub-plot which facilitates to see the difference in one or more balances according to a response categorical variance, due to a common-scale axis for all balances.

 The representation of balances is now “free” from a dendrogram structure and this presents some advantages such as ranking or selecting over the balances but also some inconveniences such as the need to check which components are in the “num” and “den” for each of the balances, which is now a moving target.

 I think that this new presentation of balance data is useful in the low-dimensional setting. Unfortunately, the interpretation of these split diagrams appears still difficult in high-dimensional space as shown in sup info example and needs an inspection of each balance sub-space. While representing proportion of variance using line thickness is innovative, it is also difficult to compare between different balances.

 The code to do this is based on ggplot2, available and easy to use and adapt to each user needs. Input is a compositional data frame and a binary partition, depending on the role of the component in the balance. Thus, the input must be generated outside of the present code, which only represents the data, which allows for flexibility, and the code is only focused on calculation and representation of pre-specified balances.

 I have some minor suggestions to improve the functionality of the code which may facilitate data interpretation:

  

- Selection: Author has added the possibility to plot multi-group boxplots. The author already mentions that this could be used for statistical testing, but it would be helpful to add an option select/highlight those balances that have statistically significant differences among groups and/or plot some statistical testing results on the right-hand-plot.
- Ranking: It looks like balances are ranked on the proportion of variance explained. However, in the provided code/examples, it is unclear whether the default ranking of the balances is the decreasing proportion of variance explained since this is not the case when the *weigh.var* option is set to TRUE. Also, it would also be useful to rank the balances according to their discrimination power over a response variable.
- With the standard balance dendrogram, when overlapping datasets, the variance for each of the subgroups could be represented (Pawlowsky-Glahn, Egozcue, Austrian Journal of Statistics,2011). I think this feature is lost in this representation.

 

 The manuscript is well written and easy to follow by the expert reader. I’d like to highlight some minor points.

- The author states that these diagrams can accommodate high dimensional data, but it does that by focusing on sub-groups of the high dimensional data, and in the code, these sub-groups are selected previous to the *balance* function. Therefore, the proposed code/diagram can really accommodate subsets of high dimensional data.

 

 Some details that caught my attention and that may be useful for future development:

  

- The name of the main function is *balance*. It overlaps with compositions::balance and ape::balance. While this is not a hard problem it may add confusion to the function namespace in this kind of analysis. I’d consider it changing it at this stage.
- When group-based boxplotting, data points are scattered over both (or more) boxplots, it would be clearer if data points were scattered over their own group boxplot.
- In the Figure S1 example. Some balances show zero values and near-zero variances, probably due to zero over-inflation and the zero-dominance(downstream imputed) balances. This is kind of a white noise in the diagram.
- Correlation: While it is outside the scope of this work, for the sake of utility, it would be helpful, when there is a continuous response variable, to plot points within the scatter plot within a y sub-axis for each boxplot in such a way that correlation between each of the balances and the response variable is visible and also be able to highlight/select those balances.

I have read this submission. I believe that I have an appropriate level of expertise to confirm that it is of an acceptable scientific standard.

# Peer review: Referee response for version 1

**DOI:** 10.5256/f1000research.17311.r37159

**Published:** 2018-08-28

**Authors:** Vera Pawlowsky-Glahn, Juan José Egozcue

**Affiliations:** 1 Department of Computer Science, Applied Mathematics and Statistics, University of Girona, Girona, Spain; 2 Department of Civil and Environmental Engineering, Polytechnic University of Catalonia, Barcelona, Spain

We have not been able to access the R-library “balance”. Consequently, we base our comments exclusively on the text presented. This circumstance motivates the answer “partly” to some of the previous questions. Thus, we cannot evaluate how well the software performs with particular datasets or how well it is documented.

 The paper “Visualizing balances of compositional data” presents an R-package to visualize balances of compositional, high dimensional, data. The original visualization of balances was in the form of a dendrogram which represented in one figure the sequential binary partition, the mean of the corresponding balances, the variance corresponding to each balance, and a boxplot of each balance, if necessary separated by subgroup in several box-plots. A dendrogram is clearly difficult to visualize if a composition has several hundreds or even thousands of components. The tool described in the paper is thus a need in the field of compositional data analysis. The visualization strategy used can be summarized in a colloquial way by saying: “separate the dendrogram in two figures, one corresponding to the partition and grouping of parts, the other to the boxplots”. It is an interesting complement to the balance dendrogram, but we do not think it is an alternative. For high dimensional compositions it would be interesting to have three or more pictures, one corresponding to the partition, one to the box-plots, and the others to particular branches of the dendrogram, possibly summarizing groups of components by their common characteristic, if any. It would look like the three pictures depicted in Figure 1 of the paper.

 One of the issues presented is the representation of the box-plots on a common scale, which is not completely new. It has been previously used at least in two papers, namely Lovell et al. (2013) ^1^ and Pawlowsky et al. (2015) ^2^. Nevertheless, the additional features of visualizing the proportion of explained variance by the thickness of the segment covering the range and by the inclusion of the data as dots can be helpful in understanding the role of each balance. At the same time, in certain circumstances, like with high dimensional data observed in a large sample, it will probably be still difficult to visualize the mentioned features. In such a case, it might be interesting to represent the first principal balances, something already considered in the paper.

 The most useful features of compositional dendrograms are (a) the visualization of the decomposition of the total variance into contributions of each balance for one or more populations in the sample; (b) the comparison of mean values between populations; (c) identifying groups of parts as they participate in balances defined by the partition. The proposed software solves point (b) efficiently by comparing box-plots in a homogenized scale. Point (c) can be supplemented including a tool able to enumerate parts in the numerator and denominator of the balance. This is important in high dimensional compositions where the labels in the partition panel are not identifiable (example Figure 2, left panel). Point (a) is more deficiently covered. A partial solution is suggested in section 3) of the paper by colouring some segments in the partition panel according to the value of the variance. However, it seems useful to have a tool allowing alphanumerical output of ordered variances or cuts of the partition tree. For instance, if somebody is looking for linear associations of groups of taxa (Egozcue, Pawlowsky-Glahn and Gloor 2018 ^3^) detecting balances which variance is smaller than a certain threshold, it is useful to visualize those balances by colour in the partition panel, but also to get a list of the parts involved in such balances.

 We expect that the presented software, modified as suggested, becomes a useful tool for the analysis of high dimensional compositional data.

 Minor issues that need to be revised in the paper are the following:

1. Introduction
  a) Nowadays, compositions are not defined as vectors of positive measurements that sum to a given total, arbitrary or not, but as representatives of equivalence classes in the positive orthant of D-dimensional real space (Barceló-Vidal et al. 2001 ^4^; Barceló-Vidal and Martín-Fernández 2016 ^5^).
  b) The usual representative of the equivalence classes is bounded between 0 and a given constant k. This defines a subset of real space which is not a subspace. Moreover, this set has a Euclidean space structure given by the operations that define the “Aitchison geometry” (Pawlowsky-Glahn and Egozcue, 2001 ^6^). Thus, it is not adequate to say that compositional data exist in a non-Euclidean space.
  c) The description of the additive (alr) and centred (clr) log-ratio transformations as “simple” is misleading. The alr defines coordinates in an oblique basis in the above mentioned Aitchison geometry, while the clr leads to coordinates in a generating system and changes with subcompositions. Thus, results of clr components are not subcompositionally coherent. Therefore, interpretation of results is very difficult, as users tend to interpret results in terms of the component in the numerator only, not taking into account the role of the denominator. Furthermore, in many cases results obtained with the alr are not permutation invariant, something that needs to be checked for each method. One of the most striking cases is e.g. regression, where the equation itself is permutation invariant, but not so the goodness-of-fit criteria.
  d) The suggested alternative analysis in terms of simple log-ratios is also not simple at all, as they lead to the most general models, i.e. general log-contrast. The exponents involved in such a log-contrast are in general different for each part of the composition.
2. Methods
  The equation given for computing balances is not correctly described. The term |i _p_| does not describe the norm or length of the sub-composition, but the number of parts in the sub-composition.
3. Use cases
  a) The optional line width of the range of box-plots to illustrate the proportion of variance explained by a balance is not really informative in the case of high-dimensional data. In the low-dimensional case we think the dendrogram is more informative, as one can recognise easily if the balance that explains the largest proportion of variance corresponds to the first steps of the partition, involving thus a large number of parts or, on the contrary, involves only a small number of parts. This deficiency can be mitigated by colouring bars in the partition panel. For instance, plotting in red the lines corresponding to a given probability quantile of large variances and in blue for a probability quantile range of small variances.
  b) Figure 2 shows two limitations of the proposed visualisation.
  i) In the left panel it is clear which taxa are involved in each balance, but not which taxa are in the numerator, and which of those are in the denominator. Perhaps a good alternative would be to use different colours for each group, or to reorder the taxa in such a way that those in the numerator are always in the left hand side and a vertical bar indicates the dividing point.
  ii) A numeration of the balances according to the larger (smaller) explained variance would be helpful in recognising rapidly which balance is the most (the less) informative in this sense.
4. Summary
  a) It is not always true that “log-ratio transformations offer a way to transform the data into an unbounded space where the analyst can apply conventional statistical method”. For this to be true you need at least the transformation to be an isometry. For example, the alr is not an isometry, and thus conventional statistical methods should not be applied blindly.
  b) Balances is a particular case of isometric log-ratio transformation. Another example is given by general log-contrasts obtained as coordinates in compositional principal component analysis.

We have read this submission. We believe that we have an appropriate level of expertise to confirm that it is of an acceptable scientific standard.

## References

[1] LovellDPawlowsky-GlahnVEgozcueJJ: Have you got things in proportion? A practical strategy for exploring association in high-dimensional compositions. *Proceedings of the 5th International Workshop on Compositional Data Analysis, CoDaWork*.2013;

[2] Pawlowsky-GlahnVMonreal-PawlowskyTEgozcueJJ: Representation of species composition. *Proceedings of the 6th International Workshop on Compositional Data Analysis, CoDaWork*.2015;

[3] EgozcueJ: Linear Association in Compositional Data Analysis. *Austrian Journal of Statistics*.2018;47(1) : 10.17713/ajs.v47i1.689 10.17713/ajs.v47i1.689

[4] Barceló-VidalCMartín-FernándezJAPawlowsky-GlahnV: Mathematical foundations of compositional data analysis. *Proceedings of IAMG'01 - The 7th Annual Meeting of the International Association for Mathematical Geology*.2001;

[5] Barcelo-VidalCMartín-FernándezJ: The Mathematics of Compositional Analysis. *Austrian Journal of Statistics*.2016;45(4) : 10.17713/ajs.v45i4.142 10.17713/ajs.v45i4.142

[6] Pawlowsky-GlahnVEgozcueJJ: Geometric approach to statistical analysis on the simplex. *Stochastic Environmental Research and Risk Assessment*.2001;384-398
